# A chinese case of prevotella intermedia and streptococcus constellatus intracranial mixed infection

**DOI:** 10.1007/s11011-017-0142-x

**Published:** 2017-11-02

**Authors:** Shanying Mo, Liuhua Wei, Hongmou Chen, Rui Li, Shuping Li, Guolan Luo

**Affiliations:** 1 Medical Science Laboratory, the Fourth Affiliated Hospital of Guangxi Medical University, Liuzhou, Guangxi China; 2 Department of Neurosurgery, the Fourth Affiliated Hospital of Guangxi Medical University, Liuzhou, Guangxi China; 3 MRI Department, the Fourth Affiliated Hospital of Guangxi Medical University, Liuzhou, Guangxi China; 4 Department of Radiology, the Fourth Affiliated Hospital of Guangxi Medical University, Liuzhou, Guangxi China

**Keywords:** Prevotella intermedia, Streptococcus constellatus, Intracranial mixed infection, MRI, CT, China

## Abstract

Streptococcal Species is increasingly recognized as a potentially preventable emerging infection in human’s brain with high prevalence around the world. Streptococcus constellatus is one of the most common pathogens. Meanwhile, anaerobic bacteria are the rare causes for intracranial infection. To date, intracranial mixed infection caused by Prevotella intermedia and Streptococcus constellatus has not been reported. We reported a Chinese case to raise the global awareness of severity of the intracranial mixed infection. Here, we illustrated the epidemiological risk factors, clinical manifestations and outcomes of the patient. For patients who suffer from exacerbated brain infection with fetid cerebrospinal fluid, early repeated imaging is urgently needed and empiric antibiotic therapy should consider anaerobic and aerobic bacteria in these situations.

## Background

Streptococcal Species is an increasingly recognized intracranial infectious pathogen. Streptococcus constellatus has been described as the fourth common clinical cause (Chang et al. 2002).There were increasing reports of brain infection caused by anaerobic bacteria, such as Prevotella oris and Peptostreptococcus micros (Frat et al. 2004), Parvimonasmicra (Ko et al. 2015), Peptoniphilus asaccharolyticus (Okui et al. 2016) and Polymicrobial anaerobic(Llitjos et al. 2016), although anaerobic culture of cerebrospinal fluid (CSF) was not recommended(Baron et al. 2013). “Spontaneous” form and “mixed” bacterial meningitis rarely appeared in middle-aged person, except in the post-neurosurgical (post-NS) patients(Tsai et al. 2012). To date, there have not been any cases about cerebral infarction accompanied by intracranial mixed infection caused by Prevotella intermedia and Streptococcus constellatus in China.

## Case report

The patient, a 48-year-old previously healthy man, was sent to the hospital presented with no obvious cause for sudden right-sided weakness, chills and fever one day ago. Cranial CT showed the left frontal lesions with unknown cause. However, his illness was aggravating. He became unconscious and irritable, and then was rushed to the Fourth Affiliated Hospital of Guangxi Medical University for further treatment. The patient’s families denied both he and his family had a history of hypertension, diabetes, coronary heart disease and other chronic or infectious diseases like hepatitis and tuberculosis etc. The patient’s parents, siblings, and children were all physically fit, but the patient had been smoking and drinking for many years and had a history of alcoholic liver.

**Fig. 1:**
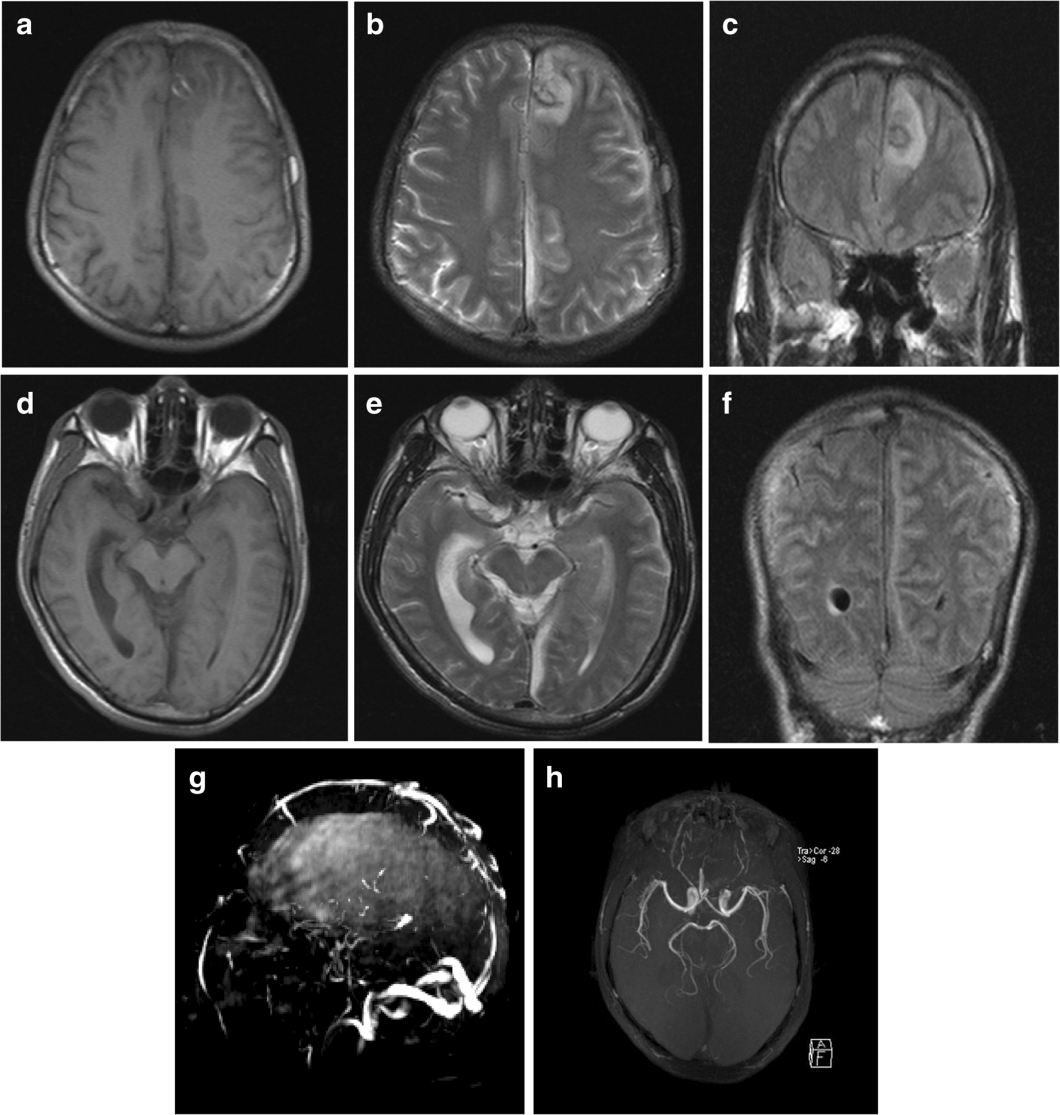
**a-f** Brain MRI revealed the left hemisphere swelled, whose cerebral sulcus and schizencephaly shallowed, especially in cerebral cortex, with patchy signal intensity which was gyriform on brain T2-weighted imaging (T2WI) and slightly high by fluid attenuation inversion recovery (FLAIR); the left frontal lobe, whose internal annular signal intensity was high on T1WI, low on T2WI and low on FLAIR was observed with patchy signal intensity that was slightly low on T1-weighted images (T1WI), slightly high on T2WI and high on FLAIR; the left falx was observed with striped signal intensity that was low on T1WI, high on T2WI and low on FLAIR. **g** MRV showed that straight sinuses were not clearly seen and superior sagittal sinus was not clear enough to be seen with rough cerebrovascular walls and focal luminal stenosis. **h** The cranial MRA showed no obvious abnormalities

On examination, he was confused and disorientated with a temperature at 40 °C. Two pairs of aerobic and anaerobic blood cultures were performed immediately. He had a high blood pressure of 163/98 mmHg and meningeal signs, with tingling eyes open, no words, no action obeyed, tingling left limb visible activity and neck resistance (+). Brain magnetic resonance imaging (MRI) revealed swelling of the left hemisphere, shallowing of sulcus and schizencephaly, abnormal lesions at the left frontal lobe and the left falx, narrowing of the left lateral ventricle due to compression and the midline structure shifted to the right (Fig. 1a-f). The cranial magnetic resonance venography (MRV) images showed abnormality in straight sinuses and superior sagittal sinus (Fig. 1g). The cranial magnetic resonance angiography (MRA) images suggested no obvious abnormalities (Fig. 1h). Results of blood tests and blood gas tests were shown in Tables 1 and 2. Results of hepatitis B virus (HBV) tests in time-resolved fluoro immune assay (TRFIA) were shown in Table 3. According to the above mentioned examinations, the patient was diagnosed with intracranial infection and started to take ceftriaxone sodium empirically.

**Table 1:** Results of blood tests

Item	Day 2	Day 3	Reference interval
White blood cell	9.51	28.28	(3.97–9.15) × 109/L
Neutrophils	9.01	25.57	(2–7.5)× 109/L
CRP	>200	>200	<10 mg/L

**Table 2:** Results of blood gas tests

Item	Day 2	Day 3–1	Day 3–2	Reference interval
PH	7.515	7.452	7.629	7.35–7.45
PCO2	24.2	30.3	13.8	35-45 mmHg
PO2	113.7	119.1	58.7	80-100 mmHg

**Table 3:** Results of HBV (TRFIA)

Item	Quantitative results	Reference interval
HBsAg	0.000	0–0.2 ng/ml
HBsAb	>640.000	0-10mIU/ml
HBeAg	0.000	0–0.5 PEIU/ml
HBeAb	0.560	0–0.2 PEIU/ml
HBcAb	5.661	0–0.9 PEIU/ml

On the second day, the patient complained of dizziness and headache without nausea. Due to unexplained cerebral infarction, enteric-coated aspirin was taken for anticoagulation and atorvastatin calcium for plaque stabilization. Lumbar puncture was performed to draw CSF. The CSF was with putrid odor and noted to be cloudy and soup-like with a protein level of 2433 mg/L, a glucose level of 0.05 mmol/L (concomitant serum glucose was 7.97 mmol/L), and 24.451 × 10^9^ leukocytes /L (80% neutrophils and 20% lymphocytes). CSF gram staining revealed gram positive cocci and gram negative bacilli. Cultured specimens were planted on the blood agar and chocolate agar for incubation at 37 °C under aerobic and anaerobic conditions regarding the smell of CSF.

**Fig. 2:**
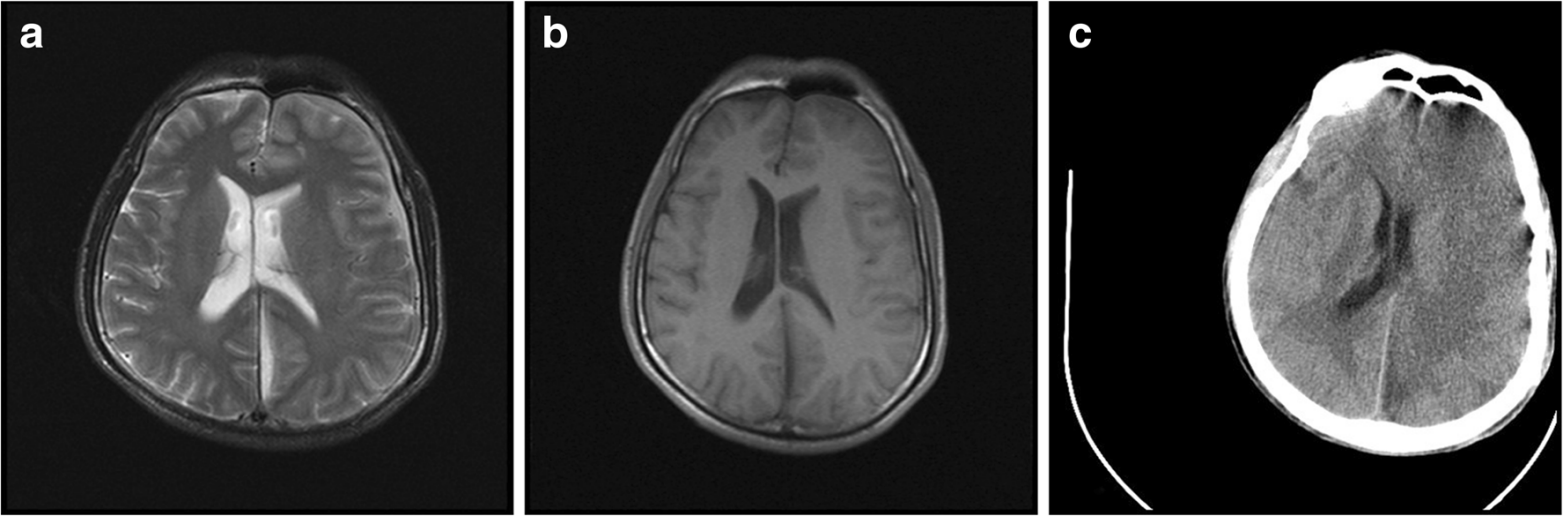
Compared with the pre-treatment MR(Fig. 2a T2WI, Fig. 2b T1WI), the post-treatment CT (Fig. 2c) revealed aggravated swelling of the left cerebral hemisphere, especially in cerebral cortex, shallowing of cerebral sulcus and schizencephaly, narrowing of the left lateral ventricle due to compression. The midline structure had shifted to the right obviously,which indicated cerebral hernia was developed

At 18:27 on the third day, blood gas test showed that type I respiratory failure, respiratory alkalosis and metabolic acidosis occurred in the patient with shortness of breath even if he was assisted by ventilator. At 22:55, the patient showed severe consciousness disorder and coma, with GCS3 points, disappeared light reflex. Brain herniation was considered, and the CT scan of head was taken immediately (Fig. 2). It showed that the patient had cerebral hernia and should receive craniotomy and decompressive craniectomy. At 23:48, bilateral mydriasis in the preparation of anesthesia appeared in the patient with his blood pressure of 30–40/10-20 mmHg and heart rate of 140 beats per minute, which had no surgical indications. Then the patient was sent back to the ward.

At 3:00 on the fourth day, the patient’s family gave up treatment and asked to be discharged, even though the patient was still in the critical condition. After that, the colonies in CSF were identified as Prevotella intermedia and Streptococcus constellatus using matrix-assisted laser desorption ionization time-of-flight mass spectrometry (MALDI-TOF MS) VITEK® MSMALDI-TOF MS (bioMériux) with a 99.9% probability. It took only four days from the symptom onset to treatment abandoning. Finally, the results of blood bacterial culture were negative.

## Discussion

Adult bacterial meningitis (ABM) is a serious infectious disease of the central nervous system (CNS)(Chang et al. 2008; Durand et al. 1993). Unlike brain abscess, it is typically a mono-microbial infection (Ruef 2000). According to the criteria in the literature(Tsai et al. 2012), the case reported was classified as the “spontaneous” form and “mixed” bacterial meningitis (Chang et al. 2000). Mixed infection was not uncommon in post-NS ABM (Lai et al. 2013), but rarely appeared in “spontaneous” form caused by anaerobic and aerobic bacteria.

Streptococcus constellatus is generally a commensal organism found in the mouth, oropharynx, and gastro intestinal tract. This organism is not only cultured from dental caries and periodontal disease but also isolated from gastrointestinal perforations, obstetric infections, brain abscesses and meningitis (Chang et al. 2002; Piscitelli et al. 1992; Roca et al. 1998; Moller et al. 1999) .

Prevotella, previously classified in the genus Bacteroides, is a genus of an obligate anaerobic gram-negative rod-shape bacterium (Shah and Collins 1990). Many species of the genus Prevotella are pathogens that cause oral diseases. Prevotella intermedia is known to cause various oral disorders such as periodontal disease (Mombelli et al. 2000) and periapical periodontitis(Jacinto et al. 2003), colonize in the respiratory tract and is associated with cystic fibrosis and chronic bronchitis (Brook and Frazier 2003) and abscesses in the head and neck spaces (Bancescu et al. 2015), as well as meningitis(Brook 2003).

Prevotella strains and Streptococcus constellatus were reported to be isolated from pus specimens in abscesses in head and neck spaces in a Romanian study (Bancescu et al. 2015). Pin-Chieh Wu et al. reported in 2014 that Prevotella brain abscesses accompanied by stroke would occur after dental extraction in a young patient (Wu et al. 2014). For the 48-year-old patient in this case, no dental extraction, dental carries or other oral diseases were reported in the case records. But their possibility as the cause to infection could not be ruled out since most adults had evidence of dental cavities (Contreras et al. 2010). On the other hand, no abscesses in head were reported in this case as well.

In a report from Pin-Chieh Wu (Wu et al. 2014), a 32-year-old healthy man with Prevotella brain abscesses and stroke following dental extraction experienced nine days of progressive headache and symptoms appeared 11 days later. Unlike the young man, the patient in this case suffered from right-sided weakness, chill and fever without any obvious causes and was immediately examined with sudden acute cerebral infarction and mixed infection caused by Prevotella intermedia and Streptococcus constellatus. The disease progression only lasted four days until his family abandoned treatment due to the exacerbation of the disease.

In a hospital-based study in Taiwan (Chang et al. 2008), diabetes mellitus (DM) was the most common underlying disease in patients with spontaneous infections, followed by liver disease, especially liver cirrhosis and alcoholism. In this study, the patient with spontaneous mixed infectious ABM had a history of HBV infection and had addiction to smoking and alcohol with years of alcoholic liver. Mixed infection was relatively few. Over a period of 10.5 years in a hospital, only 21 cases (totaling 52 strains) of mixed infection were reported and 86% of the mixed infected groups were classified as post-NS type of ABM (Tsai et al. 2012).

Consistent with previous report (Llitjos et al. 2016), the patient’s white blood cell(WBC) and protein level in CSF increased, while CSF glucose level decreased significantly with poor prognosis. ABM patients with mixed infection had a lower WBC count, lower protein and lactate concentration when compared to those with mono-microbial infection in the previous reported CSF study (Tsai et al. 2012). However, the leukocytes in patient in this case was 24.451 × 10^9^/L, which was significantly higher than those in the previous study, while the glucose showed the opposite result compared with those in the previous study (Tsai et al. 2012).

It might be a feasible way to combine gram staining with the characteristics and odor of CSF so as to identify meningitis with mixed infection of anaerobic bacteria preliminarily. *Bacteroides fragilis* concealed in an infant with *Escherichia coli* meningitis, and the apparent concealment of *B. fragilis* among *E. coli* in the CSF was possible because of their morphologic similarity; both organisms appeared as gram-negative, rod-shaped bacteria(Ganeshalingham et al. 2014). Frank pus was obtained from the *E. coli* and *B. fragilis* mixed infectious meningitis (Ganeshalingham et al. 2014). Foul smelling pus also appeared in the brain abscess due to Streptococcus spp. and Prevotella spp. (Sakamoto et al. 2009). For the patient in this study, lumbar puncture was performed to draw the soup-like CSF with putrid odor and gram staining revealed gram positive cocci and gram negative bacilli.

It might not be sufficient to use cefotaxime alone for meningitis with mixed infection with anaerobic bacteria. Antibiotic resistance was increasing not only in aerobic pathogens but also in anaerobic pathogens(Aldridge 1995). In anaerobes, production of β-lactamases is the most common mechanism of β-lactam resistance and is most frequently encountered in the *B. fragilis* group and the genus Prevotella with significant interspecies differences (Dubreuil et al. 2003). In an infant with *E. coli* and *B. fragilis* mixed infectious meningitis (Ganeshalingham et al. 2014), *B. fragilis* was confirmed resistant to ceftriaxone. In an in-vitro susceptibility test (Weintraub et al. 2016), Prevotella isolates were susceptible to metronidazole but resistant to ceftriaxone. It is well-known that metronidazole had a very good activity against most strict anaerobes, including Prevotella (Bancescu et al. 2015). Antimicrobial susceptibility testing of Prevotella spp. would take several days to complete and the delay in reporting the susceptibility results might have negative clinical implications for the patients(Sherrard et al. 2014). Metronidazole may still continue to be recommended in pyogenic diseases of head and neck but always in association with a drug active against aerobic bacteria, since such infections are usually mixed infections (Bancescu et al. 2015).

In diagnosis of cerebral infarction accompanied by meningitis with mixed infection, it is important for patients to receive imaging examination. Imaging of the brain and spine should be performed in a timely manner and reviewed specifically(Ganeshalingham et al. 2014). Brain CT scan and contrast-enhanced magnetic resonance images contributed to discovering cervical spinal epidural abscess and meningitis (Frat et al. 2004). Cranial MRA, MRI and head CT on the patient in our case showed cerebral infarction and whole brain swelling to reveal the signs of brain infection.

The patient was initially sent to the hospital for brain infarction, and then was diagnosed with intracranial mixed infection. At last, his family abandoned treatment. There might be several reasons for the exacerbation of the disease in such a short time. First, cerebral infarction aggravated; second, ceftriaxone used alone and Prevotella spp. might be resistant to ceftriaxone; third, fimbriae of Prevotella intermedia could induce hemagglutination (Leung et al. 1996), and then antibiotic resistance might accelerate the deterioration process of cerebral infarction.

Vancomycin plus a third or fourth generation of cephalosporin were the initial empiric antibiotics used in the treatment of patients with suspected ABM and the antimicrobial regimen was adjusted subsequently after the culture results were available (Tsai et al. 2012).When anaerobic bacteria was suspected to induce mixed infectious meningitis, empiric antibiotic therapy should consider anaerobic bacteria timely.

There were no previously reported risk factors expect a history of alcoholic liver and HBV infection for the patient. In this case, bacterial brain infection might appear as a cause to cerebral infarction when there was no trauma. “Spontaneous” form mixed infectious meningitis was rare, but it might be possible to occur. Anaerobic bacteria isolation was difficult, which usually required specific methods, and could be delayed (Frat et al. 2004). It should be noted that anaerobic culture should be added for the stench or cloudy CSF.

## Conclusion

This case demonstrates the possibility of cerebral infarction accompanied by mixed infectious meningitis. The clinical and laboratory indicators of ABM with mixed infection are not distinctive. In summary, the case presents the intracranial infection caused by both Prevotella intermedia and Streptococcus constellatus which is of great clinical pressure due to its fatal consequences. Therefore, it is critical to receive early repeated imaging for patients with mixed infection meningitis accompanied with cerebral infarction; empiric antibiotic therapy should consider aerobic and anaerobic bacteria infection when brain diseases occur due to unknown reasons with turbid and stench CSF. In this sense, it is important to identify and diagnose mixed infectious meningitis at early stage using CSF culture when the patient is highly suspected with specific syndromes of this infection, especially acute exacerbating meningitis.
